# Knowledge, attitudes, and experiences of ECT among psychiatric trainees and early career psychiatrists in Iran

**DOI:** 10.3389/fpsyt.2025.1555896

**Published:** 2025-04-14

**Authors:** Seyedeh Reihaneh Hosseini, Mohammadreza Shalbafan, Farnaz Ghannadi, Mahsa Boroon, Sanaz Askari, Ali Nazeri Astaneh, Mostafa Sayed Mirramazani, Cristiana Tapoi, Mariana Pinto da Costa

**Affiliations:** ^1^ Brain and Cognition Clinic, Institute for Cognitive Sciences Studies, Tehran, Iran; ^2^ Mental Health Research Center, Psychosocial Health Research Institute (PHRI), Department of Psychiatry, School of Medicine, Iran University of Medical Sciences, Tehran, Iran; ^3^ School of Medicine, Tehran University of Medical Sciences, Tehran, Iran; ^4^ Department of Psychiatry, Imam Hossein Hospital, School of Medicine, Alborz University of Medical Sciences, Karaj, Iran; ^5^ Psychoses Research Center, University of Social Welfare and Rehabilitation Sciences, Tehran, Iran; ^6^ Department of Psychiatry, Isfahan University of Medical Sciences, Isfahan, Iran; ^7^ Department of General Psychiatry, Alexandru Obregia Clinical Psychiatry Hospital, Bucharest, Romania; ^8^ Institute of Psychiatry, Psychology & Neuroscience, King’s College London, London, United Kingdom

**Keywords:** ECT, electroconvulsive therapy, attitudes, experiences, knowledge, early career psychiatrists, psychiatric trainees

## Abstract

**Objective:**

This study aimed to examine the experiences of psychiatric trainees and early career psychiatrists in Iran with Electroconvulsive Therapy (ECT).

**Methods:**

A cross-sectional survey, employing a 36-item questionnaire was conducted in Iran from March to November 2023. The survey targeted psychiatric trainees and early career psychiatrists, assessing ECT availability, training experiences, knowledge and attitudes.

**Results:**

173 responses were received. The majority of respondents were female (79.2%) and had experience in inpatient settings. About 63.0% reported ECT availability in their institutions, with 89.0% confirming the presence of specialised ECT centers within 100 km. Training in ECT was widely reported (96.5%), with 77.4% administering ECT to 10 or more patients during psychiatry training. However, only 55.5% were familiar with national ECT guidelines, and even fewer knew about international recommendations. Attitudes toward ECT were largely positive, with 86.2% agreeing on its effectiveness and 77.5% willing to recommend it to patients. ECT services were less frequently available in institutions where ECPs were employed compared to institutions where trainees were undergoing their psychiatry training. Confidence in ECT knowledge varied, with 52.6% feeling confident in their understanding, and 75.7% expressed interest in additional training.

**Conclusions:**

The study highlights a gap between ECT training and confidence among Iranian psychiatrists. Positive attitudes toward ECT and a high level of interest in further training underscore the need for enhanced educational programs and the standardisation of guidelines. Addressing stigma and policy gaps is crucial for improving ECT access and utilisation.

## Introduction

Electroconvulsive Therapy (ECT) is an effective treatment for severe psychiatric conditions, including major depressive disorder, bipolar disorder (in manic, depressive, or mixed episodes), psychotic disorders, postpartum mental disorders, and catatonia (1, 2). ECT is primarily used in two contexts: when psychiatric disorders are resistant to initial treatments (e.g. medication and psychotherapy); and in urgent or life-threatening situations, such as acute suicidality, with minimal contraindications (3–6).

ECT involves applying an electrical pulse to the scalp, inducing a seizure that typically lasts 15 to 70 seconds (7). Potential risks include complications related to general anesthesia, and oral injuries, while common side effects include post-treatment sedation, headache, nausea, muscle pain, and temporary memory loss (8). In case of memory loss, emotional and personal memories are typically preserved (9).

ECT has shown success in addressing neuroplasticity impairments by rewiring the brain, and increasing gray matter volume (10). Despite scientific support for its use, with response rates of 70% to 80% in treatment-resistant depression, ECT remains underutilised, raising concerns given the prevalence and impact of depression (6, 7). ECT is also associated with a low mortality rate in adults, approximately 2.1 per 100,000 treatments, which is lower than the mortality rate for general surgery under anesthesia (3.4 per 100,000) (11).

The introduction of modified ECT techniques in the 1950s, incorporating anesthesia, muscle relaxants, oxygenation, and monitoring, significantly improved safety and reduced side effects, making significant progress in treatment practices (7, 12). ECT can be used across diverse populations, including in pregnant women, adolescents, and the elderly to mitigate medication-related side effects (13, 14).

However, misconceptions about ECT persist, often rooted in its historical associations with inhumane practices, media portrayals and anti-psychiatry narratives, which contribute to its underuse. Patients frequently fear ECT, believing it to be violent, painful, or likely to cause memory loss or personality changes – perceptions reinforced by films and television (1). Despite efforts by professional organisations, such as the American Psychiatric Association (APA) and the Royal College of Psychiatrists (15) to provide accurate information, negative media portrayals continue to depict ECT as barbaric and cruel (16). Research conducted has refuted claims of long-term adverse effects on memory or intelligence (5, 17, 18). Nevertheless, stigma remains a significant issue, leading to social rejection, avoidance, or discrimination against those who have undergone ECT (19).

The attitudes of mental health professionals, particularly those in training, play a crucial role in shaping clinical practice (12). A German survey identified several factors associated with positive attitudes towards ECT, including professional status (physicians being more optimistic than nursing staff), feeling well-informed, and having contact with patients undergoing ECT (20). Research indicates that psychiatrists with greater knowledge and experience in ECT tend to have more positive attitudes toward its use. In the United States of America (USA), psychiatrists who referred or administered ECT expressed more positive feelings, and perceived a greater impact when involved with the treatment (21). Similarly, in South Africa, a positive relationship was observed between mental health professionals’ knowledge of ECT and their attitudes towards its use, suggesting that enhancing knowledge could improve attitudes towards the treatment (7). Physicians and healthcare professionals are essential in promoting the acceptability of ECT by educating patients and their families (19), as those who receive information from their doctors tend to have fewer fears and misconceptions (6).

In Iran, psychiatric trainees and early career psychiatrists (ECPs) face significant challenges, including the demanding nature of healthcare work, income dissatisfaction, political instability, economic sanctions, and social insecurity (22). In Iran, psychiatry training is available at over 20 medical universities, with medical doctors entering a four-year psychiatric training residency programme after a national entrance exam. Following residency, they must pass a board exam and complete a 2–5-year compulsory service as general psychiatrists before engaging in private practice (23, 24).

In Iran, ECT training is mandatory and psychiatry trainees are required to perform 100 ECT procedures before graduation, including 30 observations, 30 supervised administrations, and 40 unsupervised administrations. However, little is known in Iran on the extent that ECT is used or professionals’ attitudes towards it. This study aims to explore the context of ECT in Iran, including the availability of ECT centers, usage patterns, general attitudes towards ECT, and the accessibility of ECT educational resources.

## Methods

### Study design

This cross-sectional survey used a 36-item self-report questionnaire, administered anonymously and voluntarily.

### Data collection

The questionnaire was distributed across Iran from March to November 2023, targeting both psychiatric trainees and ECPs, defined as psychiatrists within their first five years after completing their psychiatry training. The questionnaire was disseminated via email and social media, targeting trainees from nationally recognised institutions and ECPs affiliated with the Early Career Psychiatrists Committee of the Iranian Psychiatric Association. The sample size was determined based on the available population of psychiatrists in Iran, aiming for a 20% response rate, employing a non-random, convenience sampling method.

### Instruments

Originally developed in English for an international survey (25), the questionnaire was translated into Persian by a bilingual author. The back-translation method was employed, followed by face validity assessment with input from six experts in psychiatry and psychology. Content validity was confirmed with a Cronbach’s alpha of 0.88.

The questionnaire comprised 36 questions covering: i) sociodemographic data of participants, ii) availability of ECT, ECT training experiences, and ECT guidelines within the national legal framework, and iii) attitudes towards, knowledge of, and personal interest in ECT, including viewpoints of its relevance, efficacy, safety, recommendation to patients, and associated negative perceptions. Participants were also queried about risks, contraindications, long-term harms, use in pregnant women, and their confidence in their own knowledge.

### Data analysis

Data was analysed using IBM SPSS Statistics (v. 27.0). Descriptive statistics were used to report frequencies and percentages for categorical variables. Associations between professional experience and questionnaire responses were examined using chi-square test. Odds ratios with confidence intervals were calculated for key variables (e.g., ECT availability); however, no multivariate adjustments for potential confounders were performed due to the exploratory, descriptive nature of this study. A Kruskal-Wallis test was conducted to compare confidence levels in ECT knowledge across different training types (including clinical rotations, courses/workshops, or other training formats).

## Results

### Sociodemographics

This online questionnaire was distributed to 760 psychiatrists in Iran, yielding 173 respondents (22% response rate). The sample comprised ECPs (N=89, 51.4%) and psychiatric trainees (N=84, 48.6%). The majority were female (n=137, 79.2%), married (n = 113, 65.3%), and without children (n=113, 65.3%). Over half (N=103, 59.5%) worked in inpatient psychiatric wards. Trainees were also significantly more involved in inpatient settings, whereas ECPs were more commonly working in day clinics (**Table 1**).

**Table 1:** Comprehensively outlines the sociodemographic data of the participants based on the two groups of ECPs and psychiatric trainees.

Socio-demographic variables		Count (percentage)			P-value
		Total	Psychiatric trainee	ECP	
Response rate		173 (100%)	84 (48.6%)	89 (51.4%)	
Sex	Male	36 (20.8%)	13 (15.5%)	23 (25.8%)	0.093
	Female	137 (79.2%)	71 (84.5%)	66 (74.2%)	
Marital status	Sigle	36 (20.8%)	24 (28.6%)	12 (13.5%)	0.067
	Married	113 (65.3%)	48 (57.1%)	65 (73%)	
	In a relationship	16 (9.2%)	9 (10.7%)	7 (7.9%)	
	Separated	8 (4.7%)	3 (3.6%)	5 (5.6%)	
Children	Yes	60 (34.7%)	20 (23.8%)	40 (44.9%)	0.004
	No	113 (65.3%)	64 (76.2%)	49 (55.1%)	
Workplace	Inpatient mental health center	103 (59.5%)	77 (91.7%)	26 (29.2%)	< 0.001
	Outpatient mental health center	16 (9.2%)	2 (2.4%)	14 (15.7%)	
	Day clinic	34 (19.7%)	3 (3.6%)	31 (34.8%)	
	Private practice	10 (11%)	1 (1.2%)	18 (20.2%)	
	Research center	1 (0.6%)	1 (1.2%)	0	
City	Tehran	94 (54.3%)	46 (54.8%)	48 (53.9%)	0.039
	Shiraz	54 (31.2%)	24 (28.6%)	30 (33.7%)	
	Esfahan	16 (9.2%)	7 (8.3%)	9 (10.1%)	
	Mashhad	6 (3.5%)	6 (7.1%)	0	
	Mazandaran	1 (0.6%)	1 (1.2%)	0	
	Kerman	1 (0.6%)	0	1 (1.1%)	
	Ahvaz	1 (0.6%)	0	1 (1.1%)	

### The availability of ECT, ECT training experiences, and ECT guidelines within the national legal framework

Regarding ECT availability, 109 respondents (63.0%) reported its presence in their workplace, while only 19 (11.0%) indicated the absence of a specialized ECT center within 100 km of their work institution. The vast majority (n=155, 98.8%) were confident in the use of anesthesia during ECT administration. ECT was performed in both inpatient and outpatient settings within their institutions (n=135, 78%) and nationally (n=155, 89.6%) (**Table 2**).

**Table 2:** Distribution of ECT availability, training experiences, and knowledge about guidelines among psychiatric trainees and early career psychiatrists (ECPs).

		*N (%)*
Availability of ECT specialized centers		
Is ECT available in your institution?	Yes	109 (63.0%)
	No	64 (37.0%)
Is ECT provided on an inpatient or outpatient basis in your institution?	Inpatient procedure only	33 (19.1%)
	Outpatient procedure only	5 (2.9%)
	Both	135 (78%)
Is an ECT specialized center within 100 km from your workplace?	Yes	154 (89.0%)
	No	19 (11.0%)
Is ECT provided with anesthesiology in your country?	Yes	171 (98.8%)
	No	2 (1.2%)
Is ECT provided on an inpatient or outpatient basis in your country?	Inpatient procedure only	14 (8.2%)
	Outpatient procedure only	3 (1.7%)
	Both	155 (89.6%)
Experience with ECT during training		
During your psychiatry training, was ECT training available?	Yes	167 (96.5%)
	No	6 (3.5%)
What form of ECT training was available during your psychiatry training?	Courses or workshops	52 (30.1%)
	Clinical rotation	48 (27.7%)
	Other forms	73 (42.2%)
Have you witnessed ECT being administered during your training?	Yes	172 (99.4%)
	No	1 (0.6%)
Have you administered ECT with supervision during your training?	Yes	156 (90.2%)
	No	17 (9.8%)
Have you administered ECT without supervision during your training?	Yes	130 (75.1%)
	No	43 (24.9%)
How many patients have you administered ECT to during your training?	1-2	4 (2.3%)
	3-5	10 (5.8%)
	6-9	25 (14.5%)
	More than 10	134 (77.4%)
Knowledge about ECT guidelines and national legal framework		
Are you aware of national treatment guidelines on ECT in your country?	Yes	96 (55.5%)
	No	77 (44.5)
Do patients or carers need to sign an informed consent for ECT in your country?	Yes	172 (99.4%)
	No	1 (0.6%)
Are you aware of international treatment guidelines on ECT?	Yes	37 (21.4%)
	No	136 (78.6%)

With respect to ECT training experiences, 167 respondents (96.5%) confirmed the availability of ECT training during their psychiatry training. Nearly all (n=172, 99.4%) had observed ECT administration, with 156 individuals (90.2%) noting supervised administration, and 130 (75.1%) actively performing ECT themselves independently without supervision during their training. Various training methods were reported during psychiatry training, including clinical rotations (n=48, 27.7%), courses/workshops (n=52, 30.1%), and other forms of training (n=73, 42.2%). A total of 134 respondents (77.4%) had administered ECT to 10 or more patients during their training.

Regarding national and international ECT guidelines, 96 respondents (55.5%) were familiar with national ECT treatment guidelines, whereas only 37 respondents (21.4%) were aware of international treatment recommendations. Among those aware of international guidelines, specified resources included Kaplan and Sadock’s Comprehensive Textbook of Psychiatry (26), Kaplan and Sadock’s Synopsis of Psychiatry (27), and the State of Queensland (Queensland Health) Guideline for the Administration of Electroconvulsive Therapy (2018). Nearly all respondents (n=172, 99.4%) highlighted the requirement for patients or their caretakers to sign informed consent for ECT in the country.

A significant difference was observed between psychiatric trainees and ECPs regarding the availability of ECT in their institutions (χ²= 78.25, df = 1, p<0.001). ECPs were significantly more likely than trainees to report the absence of ECT availability in their workplaces, with an odds ratio of 58.82 (CI: 17.08-202.48). No significant correlations were observed between professional experience and other Yes/No responses.

### The attitudes towards, knowledge about, and personal interest in training in ECT

#### Attitudes

The majority of respondents (n=156, 86.2%) either strongly agreed or agreed that *“ECT represents an effective treatment option”*, with a few (n=15, 8.7%) being neutral, and fewer (n=2, 1.2%) disagreeing.

Similarly, the majority (n=149, 86.1%) agreed or strongly agreed that *“ECT is lifesaving for some patients who are at risk”*, whilst some (n=18, 10.4%) were neutral, and only a few (n=6, 3.5%) disagreed.

Regarding ECT safety, over three quarters agreed (n=91, 52.6%) or strongly agreed (n = 46, 26.6%) that *“ECT is a safe treatment choice”*, while some (n=24, 13.9%) held a neutral opinion, and a few (n=12, 6.9%) disagreed.

The majority of respondents agreed (n=92, 53.2%) or strongly agreed (n=42, 24.3%) with *“Recommending ECT to their patients”*, whilst some (n=27, 15.6%), remained neutral, and fewer (n=12, 6.9%) disagreed.

Most (n=49, 28.4%) disagreed or strongly disagreed (n=101, 58.4%) that *“ECT is outdated”* whilst some (n=21, 12.1%) were neutral, and only a few (n=2, 1.2%) agreed.

Half of the participants (n=88, 50.9%) strongly disagreed, and nearly a third (n=53, 30.6%) disagreed that *“ECT is a cruel treatment”*, whilst some (n=22, 12.7%) were neutral, but a few agreed (n=8, 4.6%), or strongly agreed (n=2, 1.2%).

**Figure 1:**
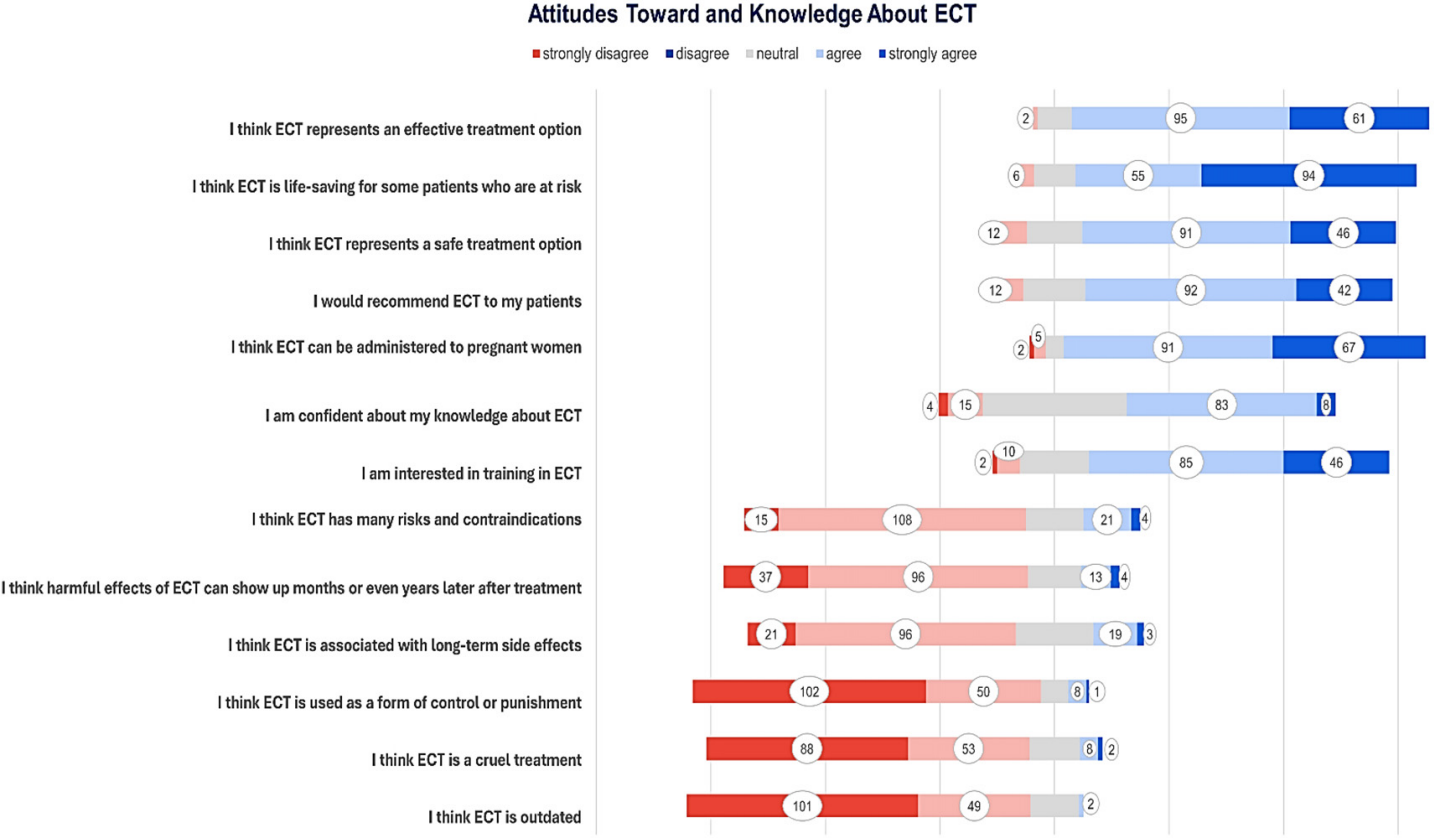
Attitudes and knowledge about ECT among respondents, displayed from strongly disagree to strongly agree.

The majority (n=152, 87.9%) were not in agreement that *“ECT as a form of control or punishment”* with most strongly disagreeing (n=102, 59%) and the rest disagreeing (n=50, 28.9%). Neutral views were held by some (n=12, 6.9%), with a few agreeing (n=8, 4.6%) or strongly agreeing (n=1, 0.6%) (**Figure 1**).

#### Knowledge

Most respondents either agreed (n=91, 52.5%) or strongly agreed (n=67, 38.7%) that *“ECT can be used on pregnant women”*, with only a few (n=8, 4.6%) neutral, disagreeing (n=5, 2.9%) or strongly disagreeing (n=2, 1.2%).

When asked whether they believe *‘ECT is associated with long-term side effects,’* most respondents disagreed (n=96, 55.5%) or strongly disagreed (n=21, 12.1%), while some remained neutral (n=34, 19.7%), agreed (n=19, 11%), or strongly agreed (n=3, 1.7%).

Almost three quarters of participants (n=133, 76.9%) were not in agreement that *“The harmful effects of ECT could manifest months or even years after treatment”*, of which most (n=96, 55.5%) disagreed or strongly disagreed (n=37, 21.4%); some remained neutral (n=23, 13.3%), agreed (n=13, 7.5%), or strongly agreed (n=4, 2.3%).

Almost half of the respondents agreed (n=83, 48%) with *“Having confidence in their knowledge about ECT”*, and some (n=8, 4.6%) strongly agreed. Nearly a third were neutral (n=63, 36.4%); some disagreed (n=15, 8.7%), or strongly disagreed (n=4, 2.3%).

**Figure 2:**
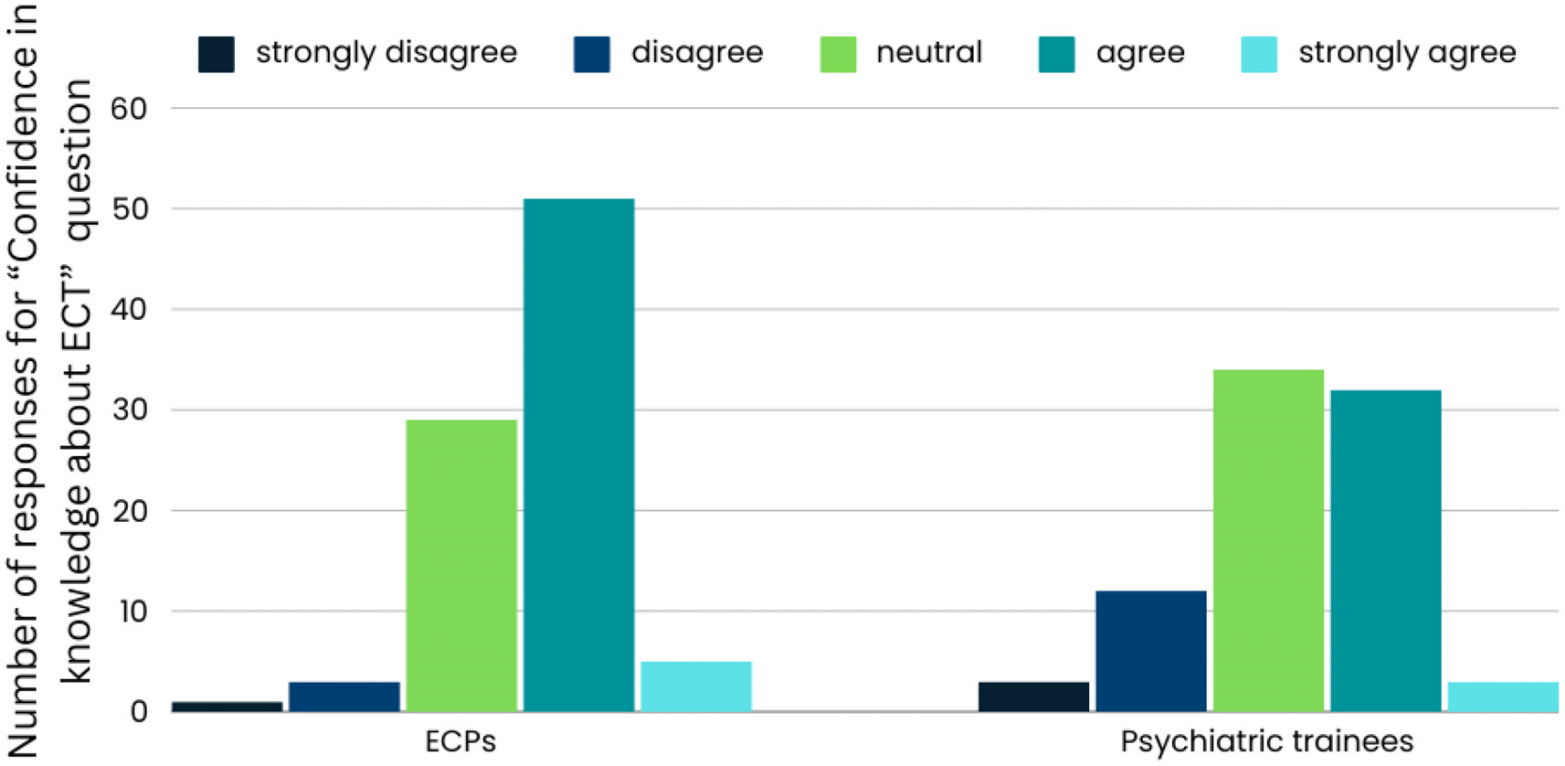
Confidence in ECT knowledge among early career psychiatrists and psychiatric trainees.

Regarding the belief that *“ECT has many risks and contraindications”* the majority (n=108, 62.4%) disagreed, some strongly disagreed (n=15, 8.7%), were neutral (n=25, 14.5%), agreed (n=21, 12.1%) or strongly agreed (n=4, 2.3%) (**Figures 1**, **2**).

#### Interest in training

The majority agreed (n=85, 49.1%) or strongly agreed (n=46, 26.6%) to have an *“Interest in receiving training in ECT”*, approximately one-third (n = 30, 17.3%) were neutral, with a few disagreeing (n=10, 5.8%) or strongly disagreeing (n=2, 1.2%).

#### Comparison

Regarding “The attitudes toward, knowledge about, and personal interest in training in ECT”, a significant difference was found in the “Confidence in their knowledge about ECT” (p = 0.016), with ECPs exhibiting higher confidence in their knowledge, and fewer disagreements. Additionally, participants who completed courses and workshops reported significantly higher confidence in their knowledge compared to those who underwent other training types (Kruskal-Wallis test, p = 0.012). The chi-square tests did not reveal any other significant differences between ECPs and psychiatric trainees for the other questions.

## Discussion

### Key findings

ECPs were significantly more likely than trainees to report the absence of ECT services at their workplaces. Several reported receiving training, observing, and administering ECT during their psychiatry training. However, familiarity with national and international guidelines was relatively low.

Overall, respondents held positive attitudes towards ECT, considering it an effective, lifesaving, and safe treatment, while rejecting the notion of it being outdated, cruel, or a form of control or punishment. Generally, respondents demonstrated a clear understanding of ECT, recognising its applicability to pregnant women and dismissing concerns about long-term side effects. Most participants expressed interest in ECT training. Whilst we distinguished between current trainees and ECPs to capture the transition from supervised residency to independent practice, this was reflected by ECPs’ significantly higher confidence in ECT knowledge and a higher likelihood of reporting the absence of ECT services in their institutions.

### Strengths and limitations

This study is the first to investigate the attitudes and knowledge of psychiatric trainees and ECPs in Iran regarding ECT. Strengths include its focus on the use of a questionnaire adapted from validated instruments, supported by a robust methodological framework. However, it has some limitations. Firstly, the low response rate (22%) and small sample size limit generalisability of the findings. Secondly, the use of self-report data may be subject to reporting bias, and the voluntary nature of the survey may lead to non-response bias. Thirdly, the recruitment strategy, predominantly via online distribution in a setting of variable internet connectivity, raises concerns about selection bias. The gender imbalance (79.2% female) may also affect findings. While this reflects the demographic distribution in psychiatric training in Iran (23, 24), response bias cannot be ruled out. Moreover, geographical limitations and cross-sectional design restrict both the general applicability and the ability to conduct chronological analysis. Additionally, the 36-item questionnaire may not cover all relevant aspects, and potential cultural barriers could affect the accuracy of responses. Finally, the anonymity of the survey precluded follow-up clarifications, leaving some responses open to misinterpretation.

### Comparison with other literature

#### Availability of ECT

Access to ECT varies significantly worldwide (3). For example, in Canada, approximately 84% of the population has convenient geographic access to ECT services (28). In Thailand, a 2022 survey revealed that 34 hospitals now offer ECT, indicating an increase from previous studies (29). In Slovenia, ECT is completely banned, although it is the only European country where this happens, according to a 2023 review (30).

In our study, most respondents acknowledged the availability of ECT services, though ECPs were more likely to report its absence in their institutions. Trainees, often based in inpatient settings or university-affiliated hospitals where typically ECT services are offered, had greater exposure compared to ECPs, who often worked in day clinics or outpatient settings with less access to ECT. This discrepancy may stem from institutional policies or regional resource variations, with some institutions lacking the necessary equipment or staff for ECT or prioritising other treatments.

In Iran, the use of the modified ECT (including anesthetics, muscle relaxants, oxygenation, and monitoring) is mandatory. However, the high cost of muscle relaxants and anesthetic drugs, along with a shortage of skilled anesthetists and restrictions in the health insurance system, limit the wide use of modified ECT (16).

#### ECT training

In 2001, the APA’s Task Force on ECT recommended that psychiatry trainees receive at least 4 hours of didactic instruction and participate in at least 10 ECT treatment procedures. However, a 2010 survey of 91 US training programs found that few met these standards: most provided less than 4 hours of lectures, and 37% indicated trainees participated in 10 or fewer ECT treatments (31, 32). In contrast, several trainees in our study reported receiving ECT training, both didactic and hands-on, with 77.4% administering ECT to 10 or more patients during their psychiatry training. Experienced clinicians also showed higher confidence in their knowledge. Similarly, a 2018 quantitative survey in Scotland found that nearly 90% of psychiatry trainees felt their ECT training was sufficient, with senior trainees rating their knowledge higher. A study conducted across Europe also revealed that ECT training is associated with a more favorable perception of its safety and efficacy among ECPs (25). This highlights strong support for ECT training and its effectiveness in building trainees’ confidence (33).

Educational resources such as videos could enhance the educational process. In France, psychiatrists and psychiatric trainees completed a questionnaire before and after watching a short educational video on ECT, which resulted in positive changes in their ECT practice (3). In Norway an interventional study developed a Virtual Reality (VR) based ECT training program, involving physicians, simulation experts, and VR developers, which received positive feedback from collaborators (32). Shifting focus from lectures to psychiatry clerkships, where trainees can observe the positive effects of ECT (33) and closely monitor patients until remission would be valuable steps (34).

ECT training is more accessible in Iran, where 96.5% of respondents reported having access, compared to 54.5% in a European survey across 30 countries (35). While countries like the UK, Portugal, Germany, and Spain offered more frequent training, access was notably lower in Romania, Greece, Albania, Latvia, and Italy.

#### National and international guidelines

Our study found that awareness of both national and international ECT guidelines was not particularly high. A 2016 survey in Italy revealed the absence of national ECT guidelines, with only 2 out of 20 regions having local standards, despite repeated government requests for national policies (34). Conversely, a 2009 survey in the Netherlands showed that 75% of institutions adhered to 14 out of 16 clinical guideline criteria, indicating high compliance with international requirements (36). Similarly, a 2012 questionnaire in Canada found that 84% of centers closely followed existing standards (37).

Globally, documents such as the APA Task Force Report, the ECT Recommendations for Health Authorities of British Columbia, and standards developed by bodies like the Royal College of Psychiatrists and the National Institute of Care and Excellence (NICE) form the foundation for ECT quality assurance. The ECT Accreditation Scheme (ECTAS) (38) is also designed to help ECT facilities in the United Kingdom and Ireland raise their standards of care. While there is no globally accepted guideline for ECT administration, these frameworks provide useful insights into ensuring ECT quality and adapting to changes in management over time (32).

#### Attitude toward and knowledge about ECT

Generally, respondents in this survey displayed positive attitudes and a thorough knowledge of ECT. Similarly, a 2015 study in Germany found that psychiatrists across various settings, including those supervising ECT therapy in hospitals, those in hospitals without ECT facilities, and those in private practice, all had a positive perception of ECT (4). Psychiatrists in Poland also exhibited more positive attitudes compared to some other Eastern European nations (39). A survey conducted in Saudi Arabia among psychiatrists and family physicians (including trainees), showed that psychiatrists had a much clearer understanding and approach to ECT compared to family physicians, suggesting a link between knowledge and attitude regarding this treatment (9). This is consistent with findings from a United Kingdom (UK) survey, where psychiatrists had the most positive attitudes and highest level of knowledge, ahead of nurses, social workers, and psychologists (14).

The finding that a small yet notable proportion of respondents perceive ECT as cruel, a form of control or punishment, and outdated is concerning. Earlier research (1) indicates that bad experiences with outdated ECT practices, when procedures were performed without adequate anesthesia and muscle relaxation, may continue to shape negative perceptions among some clinicians. Additionally, cultural attitudes and media portrayals have been shown to influence beliefs about ECT, further contributing to skepticism regarding its use (40). Interestingly, a 2011 U.S. survey revealed psychiatrists who were less knowledgeable about ECT viewed the treatment rather negatively and were less likely to refer patients (6). Among Russian psychiatrists, familiarity with ECT was more limited, and many expressed doubts about its efficacy (41). Likewise, a 2004 study among Hungarian psychiatrists revealed that 32% would decline ECT even if experiencing a psychotic depressive state. Notably, these negative perceptions were more prevalent among psychiatrists working in outpatient care settings (42).

### Impact of the findings on practice, policies and research

#### Enhancement of ECT training

Although most respondents had receiving ECT training during psychiatry training, only just over half felt confident in their knowledge. Those who attended courses and workshops reported significantly higher confidence compared to those who underwent other types of training (Kruskal-Wallis test, p = 0.012). The gap between training and confidence suggests that current training programs might not be comprehensive or practical enough. Given that 75.7% of respondents expressed interest in receiving further ECT training, future research should assess how ECT training ranks compared to other psychiatric training needs.The Iranian curriculum for psychiatry education has been revised to incorporate focused educational programs aligned with textbooks used in the US and UK, while continuously striving to integrate Iranian cultural issues and eastern psychiatric treatment modalities (23).

#### Addressing stigma and misconceptions

Despite the generally positive view within the psychiatric community in our study, some respondents regard ECT as a cruel treatment (5.8%) or believe it may be used for control or punishment (5.2%). Previous studies have identified fear and stigma as the most significant obstacles to ECT treatment access (6, 19). Since patients are likely to be less fearful of ECT and hold fewer misconceptions after receiving information from their physician (6), continuous education and awareness, both among medical professionals and the general public, become a high priority. Mental health activists should also urge cinema and the media to portray mental health conditions honestly and respectfully, in order to combat misconceptions that hinder the social inclusion of people with these conditions (43).

#### Standardisation of ECT guidelines

Our study revealed that respondents were not highly familiar with national and international ECT treatment guidelines. Given the evolving nature of ECT practices, it would be beneficial to ensure that national guidelines are reviewed and updated regularly to reflect current best practice, and disseminated amongst practitioners. Guidelines ensure a uniform approach to pretreatment assessment, premedication strategies, and the technical aspects of ECT administration (4).

### Policy recommendations

Our findings indicate that ECPs were more likely than trainees to report a lack of ECT availability in their institutions. While our study did not directly assess the need for ECT expansion, the observed differences in access suggest that further exploration of institutional barriers to ECT implementation may be warranted. Additionally, dedicated funding is necessary to maintain ECT as a viable treatment option in clinical practice.

### Future research directions

Future research should assess the long-term outcomes for patients who undergo ECT in Iran. Future research should explore whether male trainees engage differently in surveys or hold distinct perspectives on ECT. Additionally, evaluating the impact of enhanced or intervention-based training programs on clinical practice will help determine how such programs influence ECT administration. Another important area of research involves exploring the attitudes and knowledge of other healthcare professionals and the general population about ECT. Investigating the barriers to ECT utilization and the factors influencing ECT referrals could also help support broader implementation of this treatment.

## Conclusions

This study found that ECT training during psychiatry training in Iran varies, and ECT is generally available across the country. Psychiatrists are actively involved in both observing and administering ECT, and they mostly hold positive views about its effectiveness. While confidence in personal knowledge varies, there is notable interest in further training. These findings emphasize the importance of continuous education to enhance understanding and utilisation of ECT among psychiatric trainees and ECPs in Iran.

## Data Availability

The raw data supporting the conclusions of this article will be made available by the authors, without undue reservation.

## References

[1] Dowman J Patel A Rajput K. Electroconvulsive therapy: attitudes and misconceptions. J ECT. (2005) 21:84–7. doi: 10.1097/01.yct.0000161043.00911.45 15905748

[2] James BO Inogbo CF. Implementing modified electroconvulsive therapy in Nigeria: current status and psychiatrists’ attitudes. J ECT. (2013) 29:e25–6. doi: 10.1097/YCT.0b013e3182801cce 23609516

[3] Pawlak S Wathelet M Olivier F Fovet T Amad A. Impact of an educational video on the representations of electroconvulsive therapy among psychiatrists in Hauts-de-France and Occitanie. Encephale. (2021) 47:441–4. doi: 10.1016/j.encep.2021.02.019 34148645

[4] Vocke S Bergmann F Chikere Y Loh N Grözinger M. Electroconvulsive therapy as viewed by german psychiatrists: a comparison of 3 subgroups. J ECT. (2015) 31:110–3. doi: 10.1097/YCT.0000000000000208 25621540

[5] De Meulenaere M De Meulenaere J Ghaziuddin N Sienaert P. Experience, knowledge, and attitudes of child and adolescent psychiatrists in Belgium toward pediatric electroconvulsive therapy. J ECT. (2018) 34:247–52. doi: 10.1097/YCT.0000000000000489 29465501

[6] Dauenhauer LE Chauhan P Cohen BJ. Factors that influence electroconvulsive therapy referrals: a statewide survey of psychiatrists. J ECT. (2011) 27:232–5. doi: 10.1097/YCT.0b013e3181f9789c 20966767

[7] Netshilema TC Khamker N Sokudela F. Mental health professionals’ attitudes toward and knowledge about electroconvulsive therapy at Weskoppies Hospital, South Africa. Perspect Psychiatr Care. (2019) 55:201–9. doi: 10.1111/ppc.12330 30565693

[8] Rose S Dotters-Katz SK Kuller JA. Electroconvulsive therapy in pregnancy: safety, best practices, and barriers to care. Obstet. Gynecol. Surv. (2020) 75:199–203. doi: 10.1097/OGX.0000000000000763 32232498

[9] AlHadi AN AlShahrani FM Alshaqrawi AA Sharefi MA Almousa SM. Knowledge of and attitudes towards electroconvulsive therapy (ECT) among psychiatrists and family physicians in Saudi Arabia. Ann Gen Psychiatry. (2017) 16:16. doi: 10.1186/s12991-017-0139-1 28265295 PMC5331705

[10] Ousdal OT Brancati GE Kessler U Erchinger V Dale AM Abbott C. The neurobiological effects of electroconvulsive therapy studied through magnetic resonance: what have we learned, and where do we go? Biol Psychiatry. (2022) 91:540–9. doi: 10.1016/j.biopsych.2021.05.023 PMC8630079 34274106

[11] Tørring N Sanghani SN Petrides G Kellner CH Østergaard SD. The mortality rate of electroconvulsive therapy: a systematic review and pooled analysis. Acta Psychiatr Scand. (2017) 135:388–97. doi: 10.1111/acps.2017.135.issue-5 28332236

[12] Karacan FA Bağ S Karacan M Yılmaz S Yanık M. Attitudes of the mental health professionals towards unmodified and modified types of electroconvulsive therapy: a Turkey sample. Prevalence. (2021) 30:38.

[13] Ezeobele IE Ekwemalor CC Pinjari OF Boudouin GA Rode SK Maree E. Current knowledge and attitudes of psychiatric nurses toward electroconvulsive therapy. Perspect Psychiatr Care. (2022) 58:1967–72. doi: 10.1111/ppc.13016 34964509

[14] Lutchman RD Stevens T Bashir A Orrell M. Mental health professionals’ attitudes towards and knowledge of electroconvulsive therapy. J Ment Health. (2001) 10:141–50. doi: 10.1080/09638230124779

[15] (NICE) NIfCE. Guidance on the use of electroconvulsive therapy. Technol Appraisal Guidance. (2003). updated 2009.

[16] Leung CM Xiang YT He JL Xu HL Ma L Fok ML. Modified and unmodified electroconvulsive therapy: a comparison of attitudes between psychiatrists in Beijing and Hong Kong. J ECT. (2009) 25:80–4. doi: 10.1097/YCT.0b013e31817b8135 18679138

[17] Cohen D Taieb O Flament M Benoit N Chevret S Corcos M. Absence of cognitive impairment at long-term follow-up in adolescents treated with ECT for severe mood disorder. Am J Psychiatry. (2000) 157:460–2. doi: 10.1176/appi.ajp.157.3.460 10698827

[18] Ghaziuddin N Laughrin D Giordani B. Cognitive side effects of electroconvulsive therapy in adolescents. J Child Adolesc Psychopharmacol. (2000) 10:269–76. doi: 10.1089/cap.2000.10.269 11191687

[19] Wilhelmy S Rolfes V Grözinger M Chikere Y Schöttle S Groß D. Knowledge and attitudes on electroconvulsive therapy in Germany: a web based survey. Psychiatry Res. (2018) 262:407–12. doi: 10.1016/j.psychres.2017.09.015 28923432

[20] Scholz-Hehn AD Müller JC Deml R Methfessel I Zilles D Hädrich F. Factors influencing staff’s attitude toward electroconvulsive therapy: A comparison of new versus experienced electroconvulsive therapy clinics. J ECT. (2019) 35:106–9. doi: 10.1097/YCT.0000000000000544 30308568

[21] Cunningham JE Bluhm R Achtyes ED McCright AM Cabrera LY. The differential effects of psychiatrists’ and patients’ prior experiences on views about psychiatric electroceutical interventions. J Psychiatr Res. (2024) 170:11–8. doi: 10.1016/j.jpsychires.2023.12.013 PMC10872233 38101204

[22] Eissazade N Hemmati D Ahlzadeh N Shalbafan M Askari-Diarjani A Mohammadsadeghi H. Attitude towards migration of psychiatric trainees and early career psychiatrists in Iran. BMC Med Educ. (2021) 21:502. doi: 10.1186/s12909-021-02926-y 34551745 PMC8459496

[23] Eissazade N Shalbafan M Eftekhar Ardebili M Pinto da Costa M. Psychotherapy training in Iran: A survey of Iranian early career psychiatrists and psychiatric trainees. Asia Pac Psychiatry. (2021) 13:e12434. doi: 10.1111/appy.12434 33142368

[24] Eissazade N Shalbafan M Saeed F Hemmati D Askari S Sayed Mirramazani M. The impact of the COVID-19 pandemic on Iranian psychiatric trainees’ and early career psychiatrists’ Well-being, work conditions, and education. Acad Psychiatry. (2022) 46:710–7. doi: 10.1007/s40596-022-01674-5 PMC9217116 35732923

[25] Tăpoi C Alexander L de Filippis R Agorastos A Almeida D Bhatia G. Early career psychiatrists’ perceptions of and training experience in electroconvulsive therapy: A cross-sectional survey across Europe. Eur Psychiatry. (2025) 67:e86. 39801359 10.1192/j.eurpsy.2024.1798 PMC11733618

[26] Sadock BJ Sadock VA Ruiz P. Comprehensive textbook of psychiatry: lippincott Williams & wilkins Philadelphia. Wolters Kluwer, PA (2000).

[27] Kaplan HI Sadock BJ Grebb JA. Kaplan and Sadock’s synopsis of psychiatry: Behavioral sciences, clinical psychiatry: Williams & Wilkins Co. Wolters Kluwer, PA (1994).

[28] Delva NJ Graf P Patry S Gosselin C Milev R Gilron I. Access to electroconvulsive therapy services in Canada. J ECT. (2011) 27:300–9. doi: 10.1097/YCT.0b013e318222b1b8 21983755

[29] Kittayarak K Ittasakul P. Electroconvulsive therapy practice in Thailand: A nationwide survey. Neuropsychiatr Dis Treat. (2022) 18:2477–84. doi: 10.2147/NDT.S385598 PMC9635544 36338515

[30] Licht C Weirich S Reis O Kölch M Grözinger M. Electroconvulsive therapy in children and adolescents in Europe-a systematic review of the literature complemented by expert information and guideline recommendations. Eur Child Adolesc Psychiatry. (2023) 33:3389–403. doi: 10.1007/s00787-023-02248-y 37458849

[31] Menon SN Torrico T Luber B Gindoff B Cullins L Regenold W. Educating the next generation of psychiatrists in the use of clinical neuromodulation therapies: what should all psychiatry residents know? Front Psychiatry. (2024) 15:1397102. doi: 10.3389/fpsyt.2024.1397102 38812486 PMC11133724

[32] Dinwiddie SH Spitz D. Resident education in electroconvulsive therapy. J ECT. (2010) 26:310–6. doi: 10.1097/YCT.0b013e3181cb5f78 20357669

[33] Scott G Semple DM. Survey of core trainees’ Confidence in electroconvulsive therapy. J ECT. (2018) 34:113–6. doi: 10.1097/YCT.0000000000000480 29424757

[34] Buccelli C Di Lorenzo P Paternoster M D’Urso G Graziano V Niola M. Electroconvulsive therapy in Italy: will public controversies ever stop? J ECT. (2016) 32:207–11. doi: 10.1097/YCT.0000000000000301 26841302

[35] Cristiana Tăpoi LA Filippis R Agorastos A Almeida D Bhatia G Erzin G. Early career psychiatrists’ perceptions of and training experience in Electroconvulsive Therapy: a cross-sectional survey across Europe. Eur Psychiatry. (2024) 67(1):e86. 10.1192/j.eurpsy.2024.1798 PMC11733618 39801359

[36] van Waarde JA Verwey B van den Broek WW van der Mast RC. Electroconvulsive therapy in the Netherlands: a questionnaire survey on contemporary practice. J ECT. (2009) 25:190–4. doi: 10.1097/YCT.0b013e31819190b5 19190510

[37] Chan P Graf P Enns M Delva N Gilron I Lawson JS. The Canadian Survey of Standards of Electroconvulsive Therapy Practice: a call for accreditation. Can J Psychiatry. (2012) 57:634–42. doi: 10.1177/070674371205701009 23072955

[38] The ECT Accreditation Service (ECTAS). Standards for the administration of ECT. London (GB): Royal College of Psychiatrists’ Centre for Quality Improvement. London: Royal College of Psychiatrists (2020). p. 49.

[39] Antosik-Wójcińska A Gazdag G Święcicki Ł Majtczak B Rybakowski J Gosek P. Attitudes towards ECT: A survey of polish mental health professionals. Psychiatr Danub. (2021) 33:328–33. doi: 10.24869/psyd.2021.328 34795174

[40] De Schuyteneer E Dewachter B Vansteelandt K Pilato E Crauwels B Lambrichts S. Knowledge and attitudes of first- and final-year medical students about electroconvulsive therapy: the impact of media. Acad Psychiatry. (2023) 47:245–50. doi: 10.1007/s40596-023-01779-5 37058205

[41] Golenkov A Ungvari GS Gazdag G. ECT practice and psychiatrists’ attitudes towards ECT in the Chuvash Republic of the Russian Federation. Eur Psychiatry. (2010) 25:126–8. doi: 10.1016/j.eurpsy.2009.02.011 19574029

[42] Gazdag G Kocsis N Tolna J Lipcsey A. Attitudes towards electroconvulsive therapy among Hungarian psychiatrists. J ECT. (2004) 20:204–7. doi: 10.1097/00124509-200412000-00003 15591851

[43] de Filippis R Kamalzadeh L Adiukwu FN Aroui C Ramalho R El Halabi S. Mental health-related stigma in movies: A call for action to the cinema industry. Int J Soc Psychiatry. (2023) 69:1296–8. doi: 10.1177/00207640231152210 36738089

